# Macroalgal-derived alginate/wastepaper hydrogel to alleviate sunflower drought stress

**DOI:** 10.1007/s00425-023-04152-w

**Published:** 2023-05-10

**Authors:** Mohamed Gomaa, Eman S. E. Aldaby

**Affiliations:** Botany and Microbiology Department, Faculty of Science, Assiut University, Assiut, 71516 Egypt

**Keywords:** Composite hydrogel, Brown algae, Kinetics, Phosphate leaching, Phosphate adsorption, Biodegradation

## Abstract

**Main conclusion:**

Alginate/wastepaper hydrogel mitigated sunflower drought stress by increasing the water holding capacity of the soil and decreasing phosphate leaching. The hydrogel promoted sunflower growth and decreased stress related biomarkers.

**Abstract:**

There is a growing interest in the development of biodegradable hydrogels for the alleviation of drought stress on plants. A novel hydrogel based on brown algal-derived alginate and wastepaper was developed and tested as a soil supplement for sunflower growth under moderate (75% field capacity (FC)) and extreme (50% FC) water-deficit stress. The hydrogel showed fast swelling in water, which obeyed the pseudo-first order kinetics. Besides, it increased the water holding capacity of the soil and exhibited a good phosphate adsorption (37 mg PO_4_^−^ g^−1^ hydrogel after 6 days) in the soil, and more than 67% of the adsorbed phosphate was desorbed after 20 days. Thus, the phosphate leaching from the hydrogel-amended soil in a column experiment was only 2.77 mg after 4 times of over-irrigation, compared to 11.91 mg without the hydrogel. The hydrogel application promoted various root traits such as fresh and dry biomass, area, and length by > 2-, > 1.6-, > 1.35-, and > 1.3-folds under both water regimes in relation to the no-hydrogel treatments at the same conditions. Furthermore, the sunflower shoots exhibited similar proline contents to the well-watered control (100% FC), with > 50% reduction in relation to the drought-stressed plants under the same conditions. Similarly, the malondialdehyde contents were lowered by > 15%. The analysis of the antioxidant enzymes also indicated a marked reduction in the specific activities of catalase and ascorbate peroxidase under both 75 and 50% FC compared to the respective controls. Additionally, the hydrogel promoted the uptake of phosphate by sunflower roots. These results implied that the developed biodegradable hydrogel could be effectively applied as a soil additive to alleviate drought stress on crops.

**Supplementary Information:**

The online version contains supplementary material available at 10.1007/s00425-023-04152-w.

## Introduction

Water scarcity is a misfortune for plant productivity, humanity, and ecosystems. In fact, drought causes more annual loss in the agriculture sector than all the crop pathogens combined (Gupta et al. 2020). The shortage of water supplies and the growing demand for food supply for the growing population generally aggravate the effects of drought globally. Furthermore, the climatic change is another critical crisis which is anticipated to increase the severity and recurrence of drought over much of the world (Swann 2018). Generally, plants are prone to water-deficit stress due to the low water retention properties of the soil as well as the migration of water and nutrients into the underground and water loss by evaporation. A putative solution of drought stress is soil amendment which improves its water and nutrient holding capacity and reduce water percolation, thereby minimizes irrigation doses and improves plant growth (Saha et al. 2020).

Hydrogels are hydrophilic polymers that are chemically stabilized in a 3-D network structure (Fawzy and Gomaa 2021; Liu et al. 2022). Hydrogels can absorb and retain large quantities of water, thereby they can ameliorate the available water content in the soil and decrease water and nutrient losses for better crop growth (Zhu et al. 2020). The commonly used hydrogels in the agriculture are petroleum-derived monomers and polymers such as acrylic acid and polyacrylamide, which is hard to biodegrade and accumulate in the soil causing series environmental problems and biotoxicity towards humans, animals, plants and microorganisms (Chen et al. 2016; Tomadoni et al. 2020; Saha et al. 2020). Therefore, it is fundamental to develop green hydrogels based on natural renewable resources such as alginate and cellulose as a good alternative to synthetic hydrogels in agriculture.

Alginate is a linear polysaccharide in the cell wall of brown seaweeds, which consists of β-d-guluronic acid (G) and α-l-mannuronic acid (M), arranged in homo- or hetero-polymeric blocks (Gomaa 2022). Generally, alginate is non-toxic, biodegradable and forms insoluble gels when reacts with bivalent cations such as calcium (Gomaa et al. 2015). However, few attempts have been carried out to design alginate-based hydrogels for ameliorating the drought stress on plants. For instance, Tomadoni et al. (2020) used the commercially available alginate in the development of calcium alginate hydrogel with a microporous structure for the mitigation of drought stress in lettuce seedlings. In another study, a composite hydrogel based on lignosulfonate, sodium alginate and konjaku flour was fabricated and was shown to improve soil properties and the survival of tobacco plants under drought stress.

On the other hand, about 25–40% of the municipal solid waste globally is related to wastepaper (Hietala et al. 2018). Thus, it is crucial to recycle wastepaper from the economic and environmental viewpoint. Recycled wastepaper is a low-cost, sustainable and renewable source for the development of cellulose-based hydrogels. Additionally, wastepaper-based hydrogels are water insoluble and characterized by high water absorption (Fawzy and Gomaa 2020, 2021). To the best of our knowledge, no attempts have been carried out to fabricate alginate/wastepaper composite hydrogel for agricultural applications.

The aim of the present study was to utilize waste brown macroalgae and waste office paper in the development of green hydrogel (alginate/wastepaper). The developed hydrogel was tested for soil amendment to alleviate the water-deficit stress on sunflower seedlings. Various plant growth and physiological traits were carefully observed and measured to determine the effects of the hydrogel on sunflower plants. The role of the hydrogel in the minimization of water loss and phosphate leaching from soil as well as its biodegradation was also examined.

## Materials and methods

### Extraction of alginate from waste macroalgae

The brown seaweed *Sargassum latifolium* (Turner) C. Agardh was collected from the intertidal zone of the Red Sea, Egypt. The extraction of alginate from the sun-dried and milled seaweed biomass was performed by a two-stage extraction process (Fawzy and Gomaa 2021). The first stage involved the acidic pretreatment of 1.5% w/v of seaweed biomass using 2% citric acid for 2 h at room temperature and 200 rpm. The acidic pretreatment removes fucoidan and converts alginate salts into alginic acid. The second treatment involved alkaline extraction of the residual biomass (2% w/v) using 2% sodium carbonate for 3 h at 40 ºC and 200 rpm. Then, the extracted sodium alginate (SA) was collected by filtration and precipitated using 2 volumes ethanol at 4 ºC overnight. The precipitated SA was recovered by vacuum filtration and dried at room temperature. The molecular weight and the mannuronic/guluronic acid ratio of the extracted alginate was performed as described previously (Fawzy et al. 2017; Hifney et al. 2018).

### Development of alginate/wastepaper (AW) hydrogel

Waste office paper (5 g) was dissolved in 100 mL of ice-cold aqueous mixture of 7% w/v of NaOH and 12% w/v of NH_2_CONH_2_ under vigorous shaking (Fawzy and Gomaa 2020). Then acetic acid (4% v/v) was added to the mixture to precipitate cellulose (Gomaa et al. 2022). Subsequently, the regenerated cellulose (RC) from wastepaper was collected by filtration and washed several times with distilled to reach neutrality. For the preparation of hydrogel, aqueous solution of SA (4% w/v) and aqueous slurry of RC (4% w/v) were mixed to give a proportion of SA:RC of 1:1. The mixture was homogenized at room temperature under vigorous shaking (400 rpm) for 1 h. Then, the SA/RC mixture was coagulated using 1:1 (v/v) of absolute ethanol: 2% CaCl_2_. Finally, the prepared AW hydrogel was filtered and washed several times with distilled water and molded in aluminum weighing cups and was allowed to dry at room temperature. The density and porosity of the AW hydrogel was estimated as described previously (Fawzy and Gomaa 2020, 2021).

### Fourier transform-infrared spectroscopy (FT-IR) of the developed hydrogel

FT-IR spectra of the AW hydrogel was obtained using Nicolet IS 10 FT-IR spectrophotometer in the 4000–400 cm^−1^ region.

### Kinetics and thermodynamics of water absorption by the AW hydrogel

To determine the water absorption by the AW hydrogel, a known weight (*W*_0_) of the dried hydrogel was immersed in distilled water at different temperatures (25, 35, and 50 ºC). After a predetermined time, the hydrogel was removed from water and plotted against dry filter paper to remove excess unbounded water and weighed (*W*_*t*_). The swelling degree (SD, %) at each time was calculated using the following formula:

$$\mathrm{SD}=\frac{\left({W}_{t}-{W}_{0}\right)}{{W}_{0}}\times 100$$

The SD values were fitted to the following non-linear kinetic models (Fawzy and Gomaa 2020, 2021; Hifney et al. 2021):

$$\mathrm{Pseudo\,\, first}-\mathrm{order \,\,model }(\mathrm{PFO}): {q}_{t}={q}_{e}\left(1-{e}^{{k}_{1}t}\right)$$

$$\mathrm{Pseudo \,\,second}-\mathrm{order \,\,model }(\mathrm{PFO}): {q}_{t}=\frac{{k}_{2}{q}_{e}^{2}t}{1+{k}_{2}{q}_{e}}$$

where *q*_*t*_ (g g^−1^) and *q*_*e*_ (g g^−1^) are the amount of water absorbed per gram hydrogel at time *t* (min) and at equilibrium, respectively. *k*_1_ (min^−1^) and *k*_2_ (g g^−1^ min^−1^) are the water absorption rate constants for the PFO and PSO models, respectively.

Furthermore, the thermodynamic parameters such as the Gipp’s free energy (Δ*G*º, kJ mol^−1^), enthalpy (Δ*H*º, kJ mol^−1^), and entropy (Δ*S*º, J mol^−1^ K^−1^) were obtained as follows (Yang et al. 2016; Krishnan et al. 2019):

$$\Delta G^\circ = -RT\mathrm{ln}{K}_{a}$$

$${K}_{a}=\frac{{q}_{e}(\mathrm{water \,\,in \,\,gel})}{{q}_{e}(\mathrm{unabsorbed \,\,water})}$$

$$\mathrm{ln}\,{K}_{a}=\frac{\Delta S^\circ }{R}-\frac{\Delta H^\circ }{RT}$$

where *K*_*a*_ is an equilibrium constant, *R* is the universal gas constant (8.314 J mol^−1^ K^−1^), and *T* is the absolute temperature (K). The plot of ln *K*_*a*_ vs*.* 1/*T* was used to calculate the values of Δ*S*º and Δ*H*º.

### Effect of hydrogel on moisture loss from the soil

Dried soil (100 g) was amended with the AW hydrogel (2 g) in a plastic cup, then was moistened using distilled water to 100% field capacity (FC) and incubated at 25, 35, or 45 ºC. Control experiment without the hydrogel was performed similarly. The water content of the soil was determined daily by gravimetric measurements as the difference between the weight of the cup-containing soil at each time and the initial dry weight.

### Effect of hydrogel on the adsorption of phosphate from the soil

The efficiency of the AW hydrogel in adsorbing phosphate from the soil was evaluated using a soil burial test. The AW hydrogel (0.1 g) was enclosed in nylon mesh bags (0.5 mm in mesh size) and buried 3 cm under the soil (100 g) in plastic cups. Then, the soil was moistened by distilled water to reach 75% or 50% field capacity (FC). The cups were covered properly with aluminum foil and were left at 25 ºC. At predetermined time interval, the bags were removed from the soil, and the hydrogel was washed thrice with distilled water. Then, the adsorbed phosphates were released in 0.1 M HCl solution. The concentration of the desorbed phosphate was determined by spectrophotometric analysis as described by Ganesh et al. method (Ganesh et al. 2012).

### Effect of hydrogel on phosphate leaching from the soil

The efficiency of the AW hydrogel in immobilizing and minimizing phosphate leaching was determined using glass columns with 25 cm height, 3 cm width and an opening (0.3 cm) at its bottom covered with a removable plastic lid. Two layers of cotton fabrics followed by 0.3 cm layer of glass beads and another two layers of fabrics were added to the bottom of the column. Then, the column was backed with pre-dried soil (100 g) containing the hydrogel (2 g). The top of the soil was covered by a layer of glass beads trapped between two layers of fabric. At the beginning of the experiment, the soil was moistened with distilled water to reach 100% FC and were left at 25 ºC. Control experiment without the hydrogel was performed similarly. At predetermined time interval, 50 mL distilled water was added gradually to the columns and the leachates were collected. The concentration of phosphate in the leachate was determined spectrophotometrically (Ganesh et al. 2012).

### Growth experiment

Seeds of sunflower (*Helianthus annus* L. cultivar Giza 102) were surface sterilized using 4% NaOCl solution for 5 min and then washed thrice and soaked in water for one day. The growth experiments (7 seeds per plastic pot) were conducted at the greenhouse of Botany and Microbiology Department, Faculty of Science, Assiut University during Summer. The hydrogel was amended into the soil at 2% w/w and soil without the hydrogel served as a control. All the pots were irrigated with tap water at 100% FC until the appearance of the true leaves (10 days). Afterwards, the pots were divided into five treatments. The first three treatments without the hydrogel were subdivided into non-stressed (100% FC) control and drought-stressed treatments at moderate water shortage (75% FC) and extreme water shortage (50% FC). The other hydrogel-containing treatments were subjected to drought stress by maintaining the FC at 75% and 50%. The pots were weighed daily and watered to restore the required moisture by adding the calculated amount of water. The plants were harvested after 10 days of drought stress. Each treatment contained 3 pots with 5 seedlings each.

### Determination of different physiological traits of sunflower seedlings

#### Determination of free proline content

Free proline content was determined as described previously (Bates et al. 1973). Shoot samples (0.4 g) were homogenized in 3% w/v sulphosalicylic acid, and the homogenate was clarified by filtration. The filtrate (2 mL) was mixed with 2 mL ninhydrin reagent (1.25 ninhydrin in 30 mL glacial acetic acid and 20 mL 6 M phosphoric acid), and 2 mL of glacial acetic acid. The resulting mixture was heated at 100 °C for 1 h in a water bath. The reaction was terminated using an ice bath and extracted with 4 mL toluene under vigorous mixing. The absorbance of the organic toluene layer was measured at 520 nm. Proline concentration was determined using a calibration curve of *l*-proline and expressed as mg g^−1^ dry weight (DW).

#### Determination of malondialdehyde

The level of lipid peroxidation in the plant tissues was determined as 2-thiobarbituric acid (TBA) reactive metabolites, i.e., malondialdehyde (MDA). Briefly, 0.45 g shoot samples were homogenized in 2.5 mL of 0.1% w/v trichloroacetic acid (TCA), followed by centrifugation at 2400 *g* for 5 min. An aliquot of supernatant (1 mL) was mixed with 4 mL of 20% w/v TCA containing 0.5% w/v TBA. The mixture was heated at 95 °C for 30 min and cooled immediately using an ice-bath. The mixture was centrifuged at 2400 *g* for 15 min, and the absorbance of the supernatant was monitored at 450, 532 and 600 nm. The concentration of MDA was expressed as µmol g^−1^ DW using the following equation (Hodges et al. 1999):

$$\mathrm{MDA}=\frac{6.45 \times \left({A}_{532}- {A}_{600 }\right) - \left(0.56\times {A}_{450}\right)}{\mathrm{Dry \,\,weight }(\mathrm{g})}$$

#### Determination of antioxidant enzymes

Fresh leaves were homogenized in potassium phosphate buffer (50 mM, pH 7) containing ethylenediamine tetra-acetic acid (0.2 mM) and polyvinylpyrrolidone, followed by centrifugation at 4800 *g* for 20 min. The supernatant was used for the quantification of catalase, ascorbate peroxidase, and peroxidase. The protein concentration in the extract was estimated by Lowry method (Lowry et al. 1951).

Catalase (CAT, EC 1.11.1.6) activity was measured according to the method of Aebi (Aebi 1984). The reaction medium contained 2.85 mL of phosphate buffer (PB) (50 mM, pH 7), and 100 µL of the enzymatic extract. The reaction was initiated by the addition of 50 µL of 10 mM H_2_O_2_ and the decrease in absorbance at 240 nm for 60 s was recorded.

Ascorbate peroxidase (APX, EC 1.11.1.11) activity was determined as the rate of hydrogen peroxide-dependent oxidation of ascorbic acid (Nakano and Asada 1987). The reaction mixture contained 2.85 mL of PB (50 mM, pH 7) mixed with H_2_O_2_ (100 µL, 5 mM), Na_2_-EDTA (0.1 mM), ascorbic acid (50 µL, 0.5 mM) and 100 µL of enzyme extract. The oxidation of ascorbic acid was estimated from the reduction in absorbance at 290 nm for 60 s.

Peroxidase (POD) activity was determined as the rate of guaiacol dehydrogenation at 436 nm (Pütter 1974). The reaction mixture contained 3 mL PB (50 mM, pH 7) mixed with guaiacol (50 µL, 20 mM), H_2_O_2_ (30 µL, 12 mM), and 100 µl of enzyme extract.

#### Determination of soil phosphate and phosphorus uptake by sunflower

The soil collected after the harvesting of sunflower was oven-dried and used for the determination of phosphate. The available soil phosphate (Olsen’s P) was extracted from 2.5 g dried soil using 50 mL of 0.5 M NaHCO_3_ under shaking (200 rpm, 30 min) followed by filtration (Watanabe and Olsen 1965). The dried shoots and roots of sunflower were digested as described previously (Bolin and Stamberg 1944). The quantification of phosphorus in the soil extract and the plant digestate was performed spectrophotometrically (Ganesh et al. 2012). The P uptake (mg plant^−1^) was calculated as the phosphate content (mg g^−1^) of the shoot or root multiplied with the dry biomass (g) of the shoot or root.

### Biodegradation of the AW hydrogel

The biodegradation of the hydrogel was determined by a soil burial test. A known amount of AW hydrogel (*W*_0_) in a nylon mesh bag (0.5 mm in mesh size) was buried in 100 g soil in a plastic cup and 100 mL of distilled water was added, and the cups were covered with aluminum foil. After predetermined time interval, the nylon bag containing the hydrogel was removed from the soil, washed carefully using distilled water, and the collected hydrogel was oven dried at 70 ºC to obtain the residual weight (*W*_*t*_). The biodegradation of the hydrogel was calculated using the following equation:

$$\% \mathrm{Biodegradtion}=\frac{({W}_{t}-{W}_{0})}{{W}_{0}}\times 100$$

### Statistical analysis

The non-linear regression analysis of the PFO and PSO equations was obtained using the solver function in the Microsoft Excel program. The objective was to minimize the average relative error (%ARE) between the experimental and the predicted results.

$$\%\mathrm{ARE}= \frac{100}{N}\sum_{i=1}^{N}\left(\frac{\left|{q}_{t}^{\mathrm{calc}}-{q}_{t}^{\mathrm{exp}}\right|}{{q}_{t}^{\mathrm{exp}}}\right)$$

where *N* represents the number of experimental points. *q*_*t*_^calc^, *q*_*t*_^exp^ are the calculated and the experimental *q*_*t*_ values, and the index *i* identifies the experimental point.

Various growth traits of the sunflower seedlings were obtained using image analysis using ImageJ software (Schneider et al. 2012). Root area was quantified using the RIA plug-in customized for ImageJ software (Lobet et al. 2017).

Analysis of variance (ANOVA) followed by post-hoc Fisher’s least square difference (LSD) test in the GNU PSPP statistical software (V 1.6.2) was used to judge the significant differences between treatments at *p* < 0.05.

## Results

### Hydrogel characterization

The extracted alginate from the brown seaweed *S. latifolium* exhibited a viscosity average molecular weight of 1.82 × 10^5^ Da and a mannuronic/guluronic acid ratio of 1.91. A light hydrogel was prepared using the algal-derived alginate and wastepaper. The density, and porosity of the composite hydrogel at 25 ºC were 1.038 g cm^−3^, and 68.25%, respectively. While, the density, and porosity of the wastepaper alone were 1.035 g cm^−3^ and 62.70%, respectively. This implies that the incorporation of alginate into wastepaper exhibited an enhancement of the porosity with little effects on density compared to the wastepaper alone. Furthermore, the swelling degree of the AW hydrogel was 312.69%, while that of calcium alginate was 90.01%, and wastepaper was 162.42% (Fig. S1). This result indicated an enhancement of water absorption of the AW hydrogel in relation to calcium alginate and wastepaper. The prepared AW hydrogel was insoluble in water and does not alter the pH of water after swelling. Furthermore, the developed dried hydrogel was durable and stable during storage.

The FT-IR analysis of the wastepaper indicated the typical absorption bands of cellulose at ~ 1444–897 cm^−1^ (Fig. S2a). However, the band at 1641.68 cm^−1^ was related to the N−H bending of amines and C−C stretching of the aromatic ring, which was related the ink compounds (Fawzy and Gomaa 2021). The hydrogen bonded O–H and C–H stretching vibrations in the wastepaper were located at 3432.82 and 2923.20 cm^−1^, while those of alginate were located at 3443.41 and 2959.36 cm^−1^, respectively (Fig. S2a, b). The O–H vibrations in the AW hydrogel were shifted to a lower wavenumber (3406.33 cm^−1^) (Fig. S2c). The sharp bands at 1577.88 and 1418.59 cm^−1^ were assigned to the asymmetric and symmetric−COOH vibrations, respectively (Zhang et al. 2022). These bands were shifted to a higher wavenumber in the developed hydrogel (Fig. S2c). Furthermore, the bands at 1126.41 cm^−1^ and 1029.73 cm^−1^ in the alginate were related to the stretching vibrations of C−O bonds, of pyranose rings, while that at 1097.02 cm^−1^ was assigned to both C−O and C−C stretching vibrations (Fawzy et al. 2017). The vibration mode of C−O was sharp and strong in the spectrum of the AW hydrogel and located at 1118.82 cm^−1^. Additionally, the alginate spectrum showed a distinctive band of uronic acid at 945.81 cm^−1^ which attributed to C−O stretching vibrations (Fig. S2b). Similarly, the bands at 903.04 and 817.70 cm^−1^ were attributed to the α-guluronic and β-mannuronic acid (Fawzy et al. 2017).

### Kinetics and thermodynamics of water absorption by the AW hydrogel

**Fig. 1:**
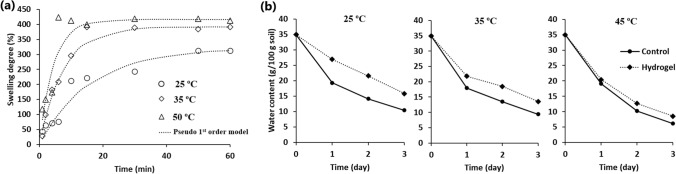
**a** Kinetics of water absorption by the AW hydrogel at different temperatures, **b** effect of hydrogel application on the water loss from the soil at different temperatures

Figure 1a depicts the swelling degree (%) of the AW hydrogel in water as a function of time and temperature. The water absorption and the swelling degree was slow at the first few minutes after contact with water. The temperature exhibited strong effects on the swelling degree of the prepared hydrogel. Consequently, the lowest swelling degree at equilibrium was 312.70% at 25 ºC and increased with the increase of temperature to reach 423.56% at 50 ºC. Furthermore, the equilibrium time of water absorption was attained in few minutes at 50 ºC (~ 6 min) compared to longer time at 35 ºC (~ 15 min) and at 25 ºC (50 min).

In general, the presence of complex network of alginate and cellulose in the composite hydrogel implied the existence of several hydrogel-water interactions. Therefore, a detailed analysis of the swelling process of the AW hydrogel is crucial for practical applications. The underlying mechanism of the swelling process of the AW hydrogel in water was determined by fitting the experimental data to kinetic models. As evident from the calculated values of *R*^2^ and % ARE, the pseudo first-order model exhibited the best fit to the experimental data at different investigated temperatures (Table S1). Based on the rate constant of the pseudo first-order equation (*K*_1_), the swelling process takes place at faster rates by increasing temperature (Table S1).

On the other hand, the investigation of the inherent energetic changes of the swelling of the developed hydrogel is also important. The negative Δ*G*º values reflected the spontaneity of the water absorption process (Table S1). Moreover, the decrease in the Δ*G*º values by increasing temperature indicated that high temperature is beneficial for the water absorption process. Furthermore, the negative Δ*H*º values implied the exothermic nature of the hydrogel swelling in water. While the positive Δ*S*º values reflected the increase in the randomness at the hydrogel/water interface during the water absorption process.

### Effect of hydrogel on moisture loss from soil

The developed AW hydrogel exhibited water retention properties when added to the soil as depicted by the decrease in moisture loss from soil compared to the soil without the hydrogel at different temperatures (Fig. 1b). After 24 h at 25 ºC, the moisture of the hydrogel-amended soil was 26.97%, which corresponds 19.37% in the control soil (Fig. 1b). Even after 3 days, the hydrogel-mixed soil retained 15.87% of its moisture compared to 10.46% under control conditions. However, the efficiency of the hydrogel to retain soil moisture was reduced by increasing the incubation temperature. Accordingly, the soil moisture in the presence of the AW hydrogel after 3 days at 35 ºC was 13.57%, which was still higher than the hydrogel-less control (9.44%) (Fig. 1b). Furthermore, about 6.17% of soil moisture was retained after 3 days at 45 ºC, but the hydrogel-mixed soil retained 8.52% of moisture content.

### Effect of hydrogel on phosphate adsorption and phosphate leaching from soil

**Fig. 2:**
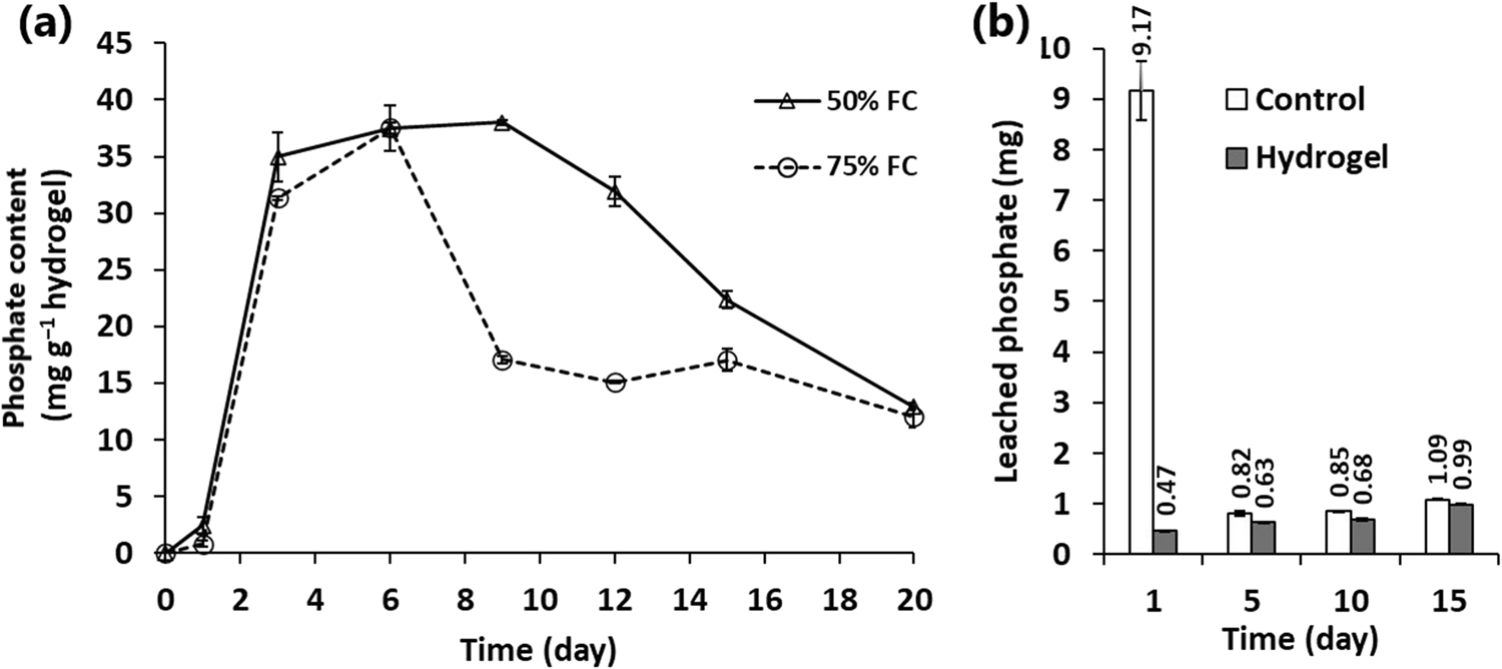
**a** Variation in phosphate content within the AW hydrogel when buried in the soil under different field capacities, **b** effect of hydrogel application on the amount of phosphate leached from the soil in four cycles of over-irrigation at different periods in a glass column experiment

The results indicated that the developed AW hydrogel had the ability to adsorb phosphorus from the soil for a certain period, followed by a desorption process (Fig. 2a). The ability of the hydrogel to adsorb and release phosphorus in the soil was highly dependent on the water status of the soil. The maximum adsorption capacity was estimated to be ~ 37 mg PO_4_^−^ g^−1^ hydrogel after 6 days of treatment. On the other hand, the process of PO_4_^−^ desorption was faster and higher in the soil contained 75% FC than that of 50% FC. However, after 20 days, the hydrogel was able to release ~ 67% of the adsorbed PO_4_^−^ regardless of the water status of the soil (Fig. 2a).

The ability of the hydrogel to adsorb phosphorus implied its fundamental role in immobilizing soil phosphate and minimizing its leaching process. This phenomenon was confirmed by a column experiment (Fig. 2b). In this experiment, in the first day of leaching, the soil without the hydrogel lost ~ 9.17 mg PO_4_^−^ compared to 0.47 mg PO_4_^−^ in the presence of hydrogel. After 5 days, the second leaching of PO_4_^−^ was reduced to ~ 0.82 mg PO_4_^−^, but it was still higher than the value observed in the presence of the hydrogel (0.63 mg PO_4_^−^). Collectively, after 4 times of over-irrigation, the soil without the hydrogel lost 11.91 mg PO_4_^−^, which corresponds 2.77 mg PO_4_^−^ in the presence of 2% AW hydrogel (Fig. 2b). These results implied that the AW hydrogel can reduce the PO_4_^−^ loss from the soil associated with over-irrigation.

### Effect of hydrogel on sunflower growth under drought stress

**Fig. 3:**
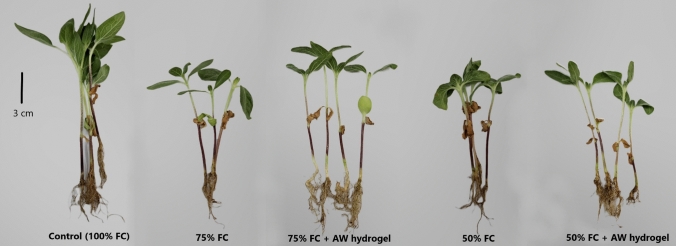
Images of sunflower seedlings at different treatments. *FC* field capacity

**Fig. 4:**
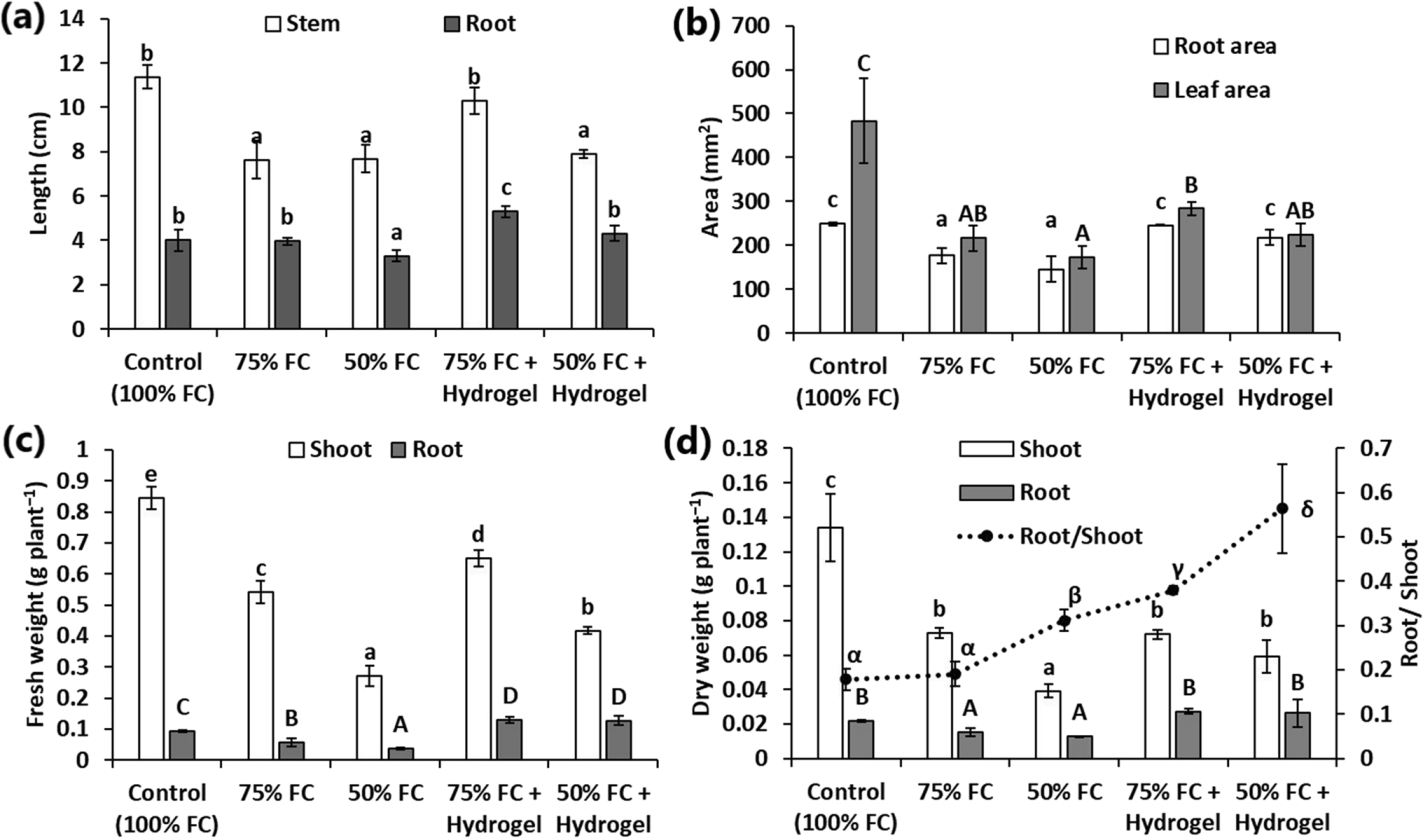
Variation in different growth traits of sunflower seedlings under different treatments. **a** stem and root length, **b** root and leaf area, **c** fresh weight of shoot and root, **d** dry weight of shoot and root and their ratio. Different letters over columns indicate significant differences at *p* < 0.05

Figure 3 depicts the variation in the growth of sunflower seedling under different treatments. The seedling height was markedly reduced by drought stress, but the responses of stems and roots were different (Fig. 4a). The stem length was significantly reduced by ~ 35% under both moderate (75% FC) and extreme water shortage (50% FC) in relation to the control (100% FC) (Fig. 4a). While, the roots were significantly shorter under extreme water shortage, but at 75% FC, the root length was statistically similar to the well-watered control (Fig. 4a). The incorporation of the hydrogel enabled the seedlings to restore their height, especially under moderate drought stress (75% FC), where the stem length was similar to the 100% FC treatment and increased by ~ 35% in relation to the no-hydrogel 75% FC treatment (Fig. 4a). Additionally, the seedlings grown in the presence of the hydrogel at 75% FC exhibited an enhancement of > 32% in the root length compared to the no-hydrogel treatments (100% and 75% FC) (Fig. 4a). In contrast, at extreme drought stress (50% FC), the AW hydrogel did not enhance stem length, but the root length was restored to the normal value at 100% FC treatment with ~ 31% increase compared to the no-hydrogel 50% FC treatment (Fig. 4a).

The root area was also influenced by drought stress. Thus, a significant reduction in root area was observed in the no-hydrogel drought-stressed conditions (Fig. 4b). Conversely, the hydrogel contributed to the restoration of the root area to their original values of the well-watered control under both 75% and 50% FC treatments (Fig. 4b). Thus, the root area in the pots amended with the hydrogel was increased by ~ 39 and 49% at 75 and 50% FC in relation to their respective no-hydrogel treatments, respectively.

The fresh and dry biomasses of both shoots and roots were markedly reduced under drought stress compared to the well-watered plants (Fig. 4c). There were more root fresh and dry weights with the AW hydrogel compared to the no-hydrogel treatments at 75% and 50% FC (Fig. 4c). Thus, more than 2-fold improvement in the fresh weight of the sunflower roots was observed in the hydrogel-containing soil. Similarly, the root dry weight exhibited > 1.6-fold enhancement by the hydrogel application compared to the no-hydrogel treatments under water deficit stress (Fig. 4c). Furthermore, the seedlings’ root in the pots amended with the AW hydrogel exhibited significantly higher fresh weights than the no-hydrogel 100% FC control (Fig. 4c). Additionally, the root dry weight under drought stress in the hydrogel-amended soil were restored to their normal values in the well-watered control (Fig. 4d).

On the other side, the application of the hydrogel enhanced the fresh biomass of the sunflower shoots at 75% and 50% FC by ~ 10% compared to the drought stressed treatments without the hydrogel (Fig. 4c). However, the 100% FC control showed the highest fresh weight of the shoots. The shoot dry weight at 75% FC exhibited non-significant variations between the hydrogel and no-hydrogel treatments (Fig. 4d). Conversely, the hydrogel application at 50% FC contributed to an enhancement of ~ 64% in the shoot dry weight compared to the no-hydrogel treatment under the same conditions (Fig. 4d). However, the shoot biomass of the hydrogel treatments was significantly lower than the well-watered control (100% FC). These variations in root and shoot biomass implied a marked variation in the root/shoot ratio. The results indicated that the seedlings grown in the hydrogel-amended soil had higher root/shoot ratios compared to the no-hydrogel treatments (Fig. 4d).

On the other side, the analysis of the area of the leaves indicated remarkable reductions in their values compared to the 100% FC treatment either in the presence or absence of the hydrogel (Fig. 4b). Therefore, the amendment of the soil with the AW hydrogel did not alter the leaf area compared to the no-hydrogel conditions of water shortage.

### Effect of hydrogel on the physiological traits of sunflower under drought stress

**Fig. 5:**
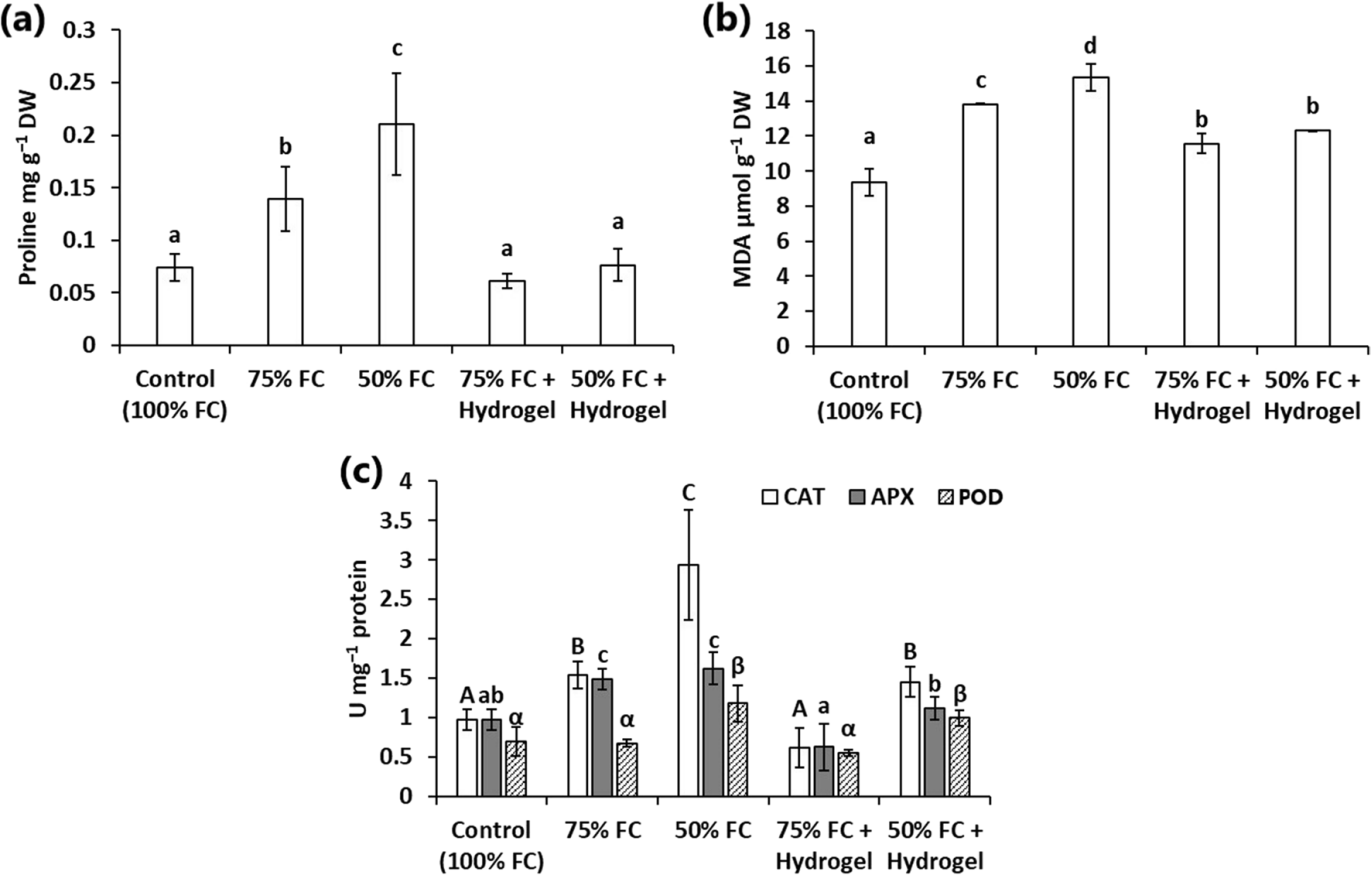
Variations in proline (**a**), malondialdehyde (MDA) (**b**), and specific activities of catalase (CAT), ascorbate peroxidase (APX), and peroxidase (POD) (**c**) under different treatments. Different letters over columns indicate significant differences at *p* < 0.05

Proline contents were highest in the sunflower shoots under drought stress in the absence of the hydrogel and increased by decreasing the water content of the soil from 75 to 50% FC (Fig. 5a). The use of the hydrogel at both moderate and extreme water shortage induced a marked reduction in the proline contents. Therefore, the application of the hydrogel reduced the proline contents of the sunflower shoots at 75% and 50% FC to their non-stressed values at 100% FC (Fig. 5a). Similarly, the MDA contents were reduced by the hydrogel amendment under both 75 and 50%FC compared to the water-deficit controls without the hydrogel (Fig. 5b). However, the MDA values observed in the presence of the hydrogel were significantly higher than the 100% FC treatment by ~ 25%.

On the other side, APX and CAT activities exhibited significant higher values in the drought-stressed shoots of sunflower grown without the hydrogel at both 75% and 50% FC compared to 100% FC treatment (Fig. 5c). The use of the AW hydrogel induced a remarkable reduction of the CAT and APX activities. Therefore, the APX activity of the sunflower grown either at moderate or extreme water shortage in the presence of the AW hydrogel was statistically similar (*p* > 0.05) to the non-stressed control (100% FC) (Fig. 5c). Similarly, the application of the hydrogel at 75% FC contributed to a marked reduction in the CAT activity compared to the no-hydrogel treatment, and the observed values showed non-significant variation to the 100% FC treatment (Fig. 5c). Additionally, the hydrogel amendment at 50% FC induced ~ 50% lower CAT activity in the sunflower than the same treatment without the hydrogel, but its values were still higher than the 100% FC treatment. On the other side, the POD activities exhibited elevated values only under 50% FC treatment either in the presence or absence of the hydrogel (Fig. 5c).

### Effect of hydrogel on soil P content and P uptake by sunflower under drought stress

**Fig. 6:**
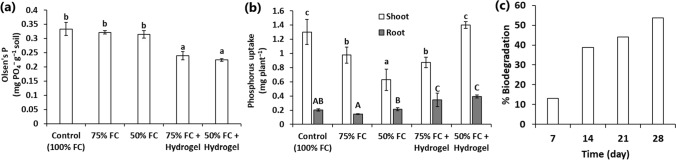
**a** Effect of hydrogel application on the amount of extractable phosphate from the soil. **b** Effect of hydrogel application on phosphorus uptake by sunflower seedlings under different treatments. **c** Biodegradation of the AW hydrogel as a function of time. Different letters over columns indicate significant differences at *p* < 0.05

The extractable soil phosphorus (Olsen’s P) contents were analyzed in the soil after sunflower harvesting. The results indicated a significant reduction in the extractable soil phosphates in the soil amended with the hydrogel in relation to the treatments without the hydrogel (Fig. 6a). This result further confirmed the ability of the hydrogel to adsorb and immobilize phosphorus in the soil. Accordingly, it is fundamental to identify the interference of the hydrogel on the phosphorus uptake by sunflower seedlings. The phosphorus uptake (mg plant^−1^) of the sunflower roots was promoted by the hydrogel amendment and reached 0.34 ± 0.1 and 0.39 ± 0.02 at 75% and 50% FC, respectively (Fig. 6b). These values were > 70% higher than all the non-hydrogel containing treatments (100%, 75%, and 50% FC). On the other side, only at 50% FC, the hydrogel treatment exhibited an elevated shoot P uptake in relation to the same treatment without the hydrogel, and the values were statistically similar to the 100% FC condition (Fig. 6b).

### Biodegradation of the AW hydrogel

The soil burial test indicated that the AW hydrogel is vulnerable to biodegradation with continuous mass loss over time. Thus, about 50% of the AW hydrogel was biodegraded after 28 days, which reflected good biodegradability.

## Discussion

Alginate derived from the waste biomass of the macroalga *S. latifolium* and waste office paper were utilized as cost-effective and eco-conscious materials for the preparation of hydrogel for agricultural applications. The FT-IR spectrum of the AW hydrogel showed a distinct shift in the O–H vibration to the lower wavenumber compared to that of alginate and cellulose, which was related to the interactions between cellulose and alginate chains through hydrogen bonding (Fawzy and Gomaa 2020). The developed AW hydrogel exhibited enhanced porosity compared to wastepaper. This result was mainly related to the reorganization of cellulose chains during regeneration using NaOH/urea solution as well as the formation of new interactions with alginate to form a 3-D porous network structure (Parajuli et al. 2020). Furthermore, the FT-IR analysis indicated the presence of several hydrophilic groups such as hydroxyl and carboxyl groups in the AW hydrogel, which can attach to water molecules by hydrogen-bonding (Saha et al. 2020). The mechanism of water adsorption by the AW hydrogel obeyed the pseudo-first order model. Accordingly, the water adsorption process was mainly controlled by the mass transfer process due to the difference between the concentration of water between the solution and the hydrogel (Hifney et al. 2021; Fawzy and Gomaa 2021). This mechanism confirms the ability of the AW hydrogel to absorb and release the water molecules as a function of osmotic pressure difference between the soil and the polymer network. Furthermore, the AW hydrogel exhibited higher swelling degrees at higher temperatures, which was attributed to the fast relaxation of the polymer networks and the higher diffusion of water molecules as a result of the increased entropy of the solution. The higher water uptake of the AW hydrogel by increasing temperature contradicts the behavior of several bio-based hydrogels (Abdel-Raouf et al. 2018; Durpekova et al. 2020). Additionally, Sharma et al. (2014) observed a reduction in the water holding capacity of calcium alginate nanoparticles by increasing temperature beyond 25 ºC, which was related to the weakening of the van der Walls forces between the alginate particles and the water molecules. In general, polymers with higher solubility at higher temperatures show an increase in their swelling ratio by increasing temperature (Tomadoni et al. 2019). The predominant hydrophilic interactions and the thermal expansion of the AW hydrogel in the present study reflected a direct dependence of the swelling degree on temperature. In fact, the behavior of the AW hydrogel is advantageous to allow more water absorption and improve water use efficiency in regions with hot weather. Therefore, it is important to evaluate the efficiency of the AW hydrogel on minimizing the moisture loss from soil at different temperatures. The results of the present study clearly demonstrated that mixing the AW hydrogel at 2% w/w delayed the moisture loss from the soil at 25, 35, and 45 ºC. Although, the efficiency of the AW hydrogel decreased by increasing temperature, the delay in moisture loss from soil generally provides more favorable conditions for plant growth and increase their survival under water-deficit stress.

On the other hand, the thermodynamic analysis demonstrated negative Δ*G*º values which indicated that the water absorption process by the AW hydrogel is spontaneous and thermodynamically favorable (Fawzy and Gomaa 2020). The decrease in the Δ*G*º values by increasing temperature reflected the better water uptake at higher temperature. Additionally, the Δ*G*º values fall within the range of physisorption (− 20–0 kJ mol^−1^), which implied that the AW hydrogel absorb water by physical mechanism through weak interactions such as hydrogen bonding and van der Walls forces (Krishnan et al. 2019). Moreover, the negative Δ*H*º values manifested that the water absorption process is exothermic. The thermodynamic behavior of the AW hydrogel resembles that of polyacrylamide hydrogel coated sand (Krishnan et al. 2022), but contradicts the behavior of polyacrylamide/graphene hydrogel (Krishnan et al. 2019).

Sunflower growth and biomass production were limited by drought stress, but the application of the AW hydrogel mitigated this effect. Most notably, various root attributes such as length, fresh and dry weight and area were markedly enhanced under water shortage conditions in the presence of the hydrogel. Generally, the decrease in root length and root biomass is the main consequence of drought stress in many plants (Zhou et al. 2018). The improvement of various root traits is generally important to enhance plant tolerance to water-deficit stress (Comas et al. 2013). These observations are interesting and implied that the hydrogel may enhance sunflower growth under both water regimes by promoting soil water status. In fact, the important role of hydrophilic polymers to enhance plant tolerance to drought stress was attributed to the supplementary water absorbed from these polymers, which improved the available water content under water-deficit conditions (M’barki et al. 2019). Accordingly, the sunflower roots could pick up water from the AW hydrogel as well as the promotion of root traits improved water uptake under water-limited conditions. The remarkable enhancement of root fresh weight compared to the well-watered control in the current study was mainly attributed to the increased water status of the hydrogel-amended soil. This in turn delays the negative effects of water deficit-stress on plants and increases their survival with potential reduction in irrigation doses.

On the other hand, significant enhancement of shoot fresh biomass was observed in pots amended with the AW hydrogel at both 75 and 50% FC compared to the respective no-hydrogel treatments at the same condition. This result implied that the increase in the water content of the sunflower shoot as a consequence of the released moisture from the hydrogel. However, significant enhancement of shoot dry biomass was observed only at 50% FC compared to the hydrogel-less control cultivated at the same FC. Conversely, the non-stressed control cultivated under 100% FC exhibited elevated shoot fresh and dry biomasses compared to other treatments. Moreover, the enhancement of root biomass with simultaneous decrease in shoot biomass induced higher root: shoot biomass ratio in the hydrogel-amended pots.

The significant enhancement of sunflower shoot and root length in pots amended with AW hydrogel at 75% FC as well as the increase in root length at 50% FC in relation to stressed controls is consistent with the effects of other natural hydrogels. For instance, Mazloom et al. (2020) observed an increase of the height of maize plants cultivated under 90, 65, and 45% FC in the presence of lignin-based hydrogel. In another study, Parvathy et al. (2014) reported an improved shoot length of chili plants cultivated in cassava starch-mixed soil and irrigated every 3-days in relation to the no-hydrogel control. Generally, the improvement of plant height may be related to that the hydrogels releases water in a synchronous manner with the biological activities that stimulates cell division and elongation (Mazloom et al. 2020).

Proline is one of the fundamental compatible solutes which are commonly detected in environmentally stressed plants. Thus, elevation of proline concentrations is a reliable indicator of drought stress level in plants (Gupta et al. 2020). Therefore, sunflower seedlings grown at 75% and 50% FC exhibited ~ 1.9- and 2.8-fold increase in the proline contents compared to the normal conditions, respectively. Conversely, the application of the hydrogel restored the proline contents of the water-stressed sunflower seedlings to the same contents of non-stressed control. Accordingly, the decrease in proline contents seems to be related to the protection of sunflower seedlings from drought stress and indicated that plants cultivated with the AW hydrogel were less stressed than the control plants cultivated at 75 and 50% FC. Similar observations were reported previously for the use of lignosulfonate/sodium alginate/konjaku floor hydrogel for tobacco growth under drought stress (Song et al. 2020). On the other side, water-shortage stress induces the production and accumulation of various reactive oxygen species (ROS), which in turn cause peroxidation of membrane lipids and releases malondialdehyde (MDA). Thus, the increase in MDA contents have been regarded as an effective indicator to assess the degree of cell membrane damage in relation to water-deficit stress (Kenawy et al. 2018). Accordingly, a linear increase in MDA contents with increasing drought level was observed in sunflower seedlings without hydrogel amendment. The mitigation of drought stress by the AW hydrogel application was reflected by the remarkable decrease in the MDA contents compared to the no-hydrogel treatments at both 75% and 50% FC. Similar findings were reported with different hydrogels (Kenawy et al. 2018, 2022), which implied that hydrogels could preserve membrane integrity by promoting the hydraulic status in the rhizosphere under water shortage and reducing the chances of ROS production. Moreover, various antioxidant enzymes such as CAT, POD, and APX are overexpressed in the stressed cells to scavenge ROS. Thus, the levels of CAT, and APX were increased in sunflower seedlings irrigated with 75 and 50% FC in the absence of the hydrogel in relation to normal control (100% FC). The application of the AW hydrogel in the present study markedly reduced CAT and APX activities, which further supports the success of the AW hydrogel in drought stress alleviation in sunflower. In accordance with this observation, Nazarli et al. (2011) reported a reduction in CAT, and APX in sunflower by the application of synthetic hydrogel. The levels of POD were also increased in drought-stressed sunflower in the 50% FC treatment only. However, the application of the AW hydrogel did not significantly reduce the POD activity at 50% FC. The non-significant changes in the POD activity in the 75% FC treatment in relation to the 100% FC control indicated that sunflower relies on CAT and APX more than POD in the elimination of ROS.

In general, the positive effects of the AW hydrogel on plant growth under water-deficit conditions may be related to the presence of alginate. The biodegradation of the alginate in the AW hydrogel by soil microorganisms results in the formation of oligo-alginates, which can be absorbed by the sunflower roots. The absorbed oligo-alginate can act as a bio-stimulant for plant growth and increase the plant response to environmental stress (Salachna et al. 2018). Zhang et al. reported an enhancement of various root traits such as length, tips, volume, fresh weight, absorbing area and activity in Chinese cabbage in the hydroponic cultures treated with alginate oligosaccharides (Zhang et al. 2009). Accordingly, the developed AW hydrogel may benefit sunflower growth by increasing the available water content in the soil and also by releasing oligo-alginates through natural biological degradation. The release of non-phytotoxic and bio-stimulant compounds by the biodegradation of the AW hydrogel could extend their function in the soil even after biodegradation. In contrast, the commonly used superabsorbent acrylate-based hydrogels in agriculture have been reported to release acrylic acid and Na^+^, which have detrimental effects on plant growth and are phytotoxic (Chen et al. 2016). Moreover, the biodegradation of the AW hydrogel in the soil can play a crucial role in improving the soil microbiota, which in turn has a fundamental role in nutrient recycling and plant development. Similarly, Tomadoni et al. reported an increase in the microbial community of the alginate-mixed soil, which supported the growth of lettuce plants under drought stress (Tomadoni et al. 2020).

Generally, drought stress can adversely influence the absorption and translocation of nutrients from soil, which in turn reduce plant growth and biomass production. However, the present study indicated that the AW hydrogel can provide the plant with nutrient and water due to its predominant hydrophilic groups. The AW hydrogel exhibited a natural adsorption and desorption process for soil phosphates. The marked enhancement of root P uptake in the pots amended with the AW hydrogel compared to the no-hydrogel controls implied the important role of the AW hydrogel in the absorption of phosphate by sunflower roots. Similarly, Nassaj-Bokharaei et al. (2021) reported an improved P uptake by tomato plants cultivated with starch/natural char nanoparticles hydrogel under drought stress. With a higher nutrient accumulation, the increased root biomass and development under water-deficit conditions could be explained. In general, the balance between nutrient and water availability and root development has fundamental effects on the tolerance of plants to water-shortage stress (Nassaj-Bokharaei et al. 2021). Accordingly, the controlled release of the adsorbed phosphate by the AW hydrogel could help sunflower plants to survive under water-deficit conditions. Moreover, nutrient loss from soil by leaching and over-irrigation is a substantial problem, with about 80–90% of P, 40–70% N, and 50–70% K from fertilizers is wasted in the environment and not absorbed by crops (Tan et al. 2021). The present study indicated that the application of AW hydrogel can save about 8.7 mg PO_4_^−^ and 9.14 mg PO_4_^−^ after the first and fourth leaching experiment compared to the amount lost from the hydrogel-less soil. Accordingly, the application of the AW hydrogel could reduce the dosage of phosphate fertilizers and their recurrence for optimum soil health.

## Conclusion

A low-cost, environmentally benign, and sustainable hydrogel was developed in the present study based on algal-derived alginate and wastepaper as a potential solution to mitigate water scarcity in cultivated soils. The hydrogel showed good swelling properties in water, which increased by increasing temperature. The AW hydrogel increased the available water content in the soil. Consequently, the growth of the sunflower seedlings was less affected by water shortage in the pots amended with the hydrogel. Therefore, various root traits such as fresh and dry biomass, length and area were promoted in the presence of the hydrogel in relation to the control treatments under the same conditions. Analysis of physiological indicators of drought stress, such as proline, malondialdehyde, and antioxidant enzymes revealed that the seedlings cultivated in pots containing the hydrogel were less stressed compared to the no-hydrogel controls subjected to water-deficit stress. Moreover, the developed hydrogel exhibited a marked role in adsorbing and desorption phosphates in the soil. This behavior enabled the hydrogel to control the phosphate leaching from the soil and increase its uptake by the sunflower roots. Moreover, further experiments in the field aimed at testing concentrations of the hydrogel and their effects on various crops would be required to better acknowledge its application in the agriculture.

### *Author contribution statement*

MG: conceptualization, validation, investigation, methodology, formal analysis, writing—original draft, writing—review and editing. ESEA: validation, investigation, methodology, writing—review and editing.

## Supplementary Information

Below is the link to the electronic supplementary material.

Supplementary file1 (DOCX 184 KB)

## Data Availability

The datasets used and/or analyzed during the current study are available from the corresponding author on reasonable request.
