# Supplementary material for: Macroalgal-derived alginate/wastepaper hydrogel to alleviate sunflower drought stress
Source: Planta. 2023 May 10;257(6):112. doi: 10.1007/s00425-023-04152-w (PMC10172250; doi:10.1007/s00425-023-04152-w)
Supplement: Supplementary file 1 — Supplementary file1 (DOCX 184 KB) [file 425_2023_4152_MOESM1_ESM.docx]

**Supporting Information**

**Macroalgal-derived alginate/wastepaper hydrogel to alleviate sunflower drought stress**

**Mohamed Gomaa*, Eman S. E. Aldaby**

**Botany & Microbiology Department, Faculty of Science, Assiut University, 71516, Assiut, Egypt**

*** Corresponding author**

**e-mail: m_gomaa@aun.edu.eg**


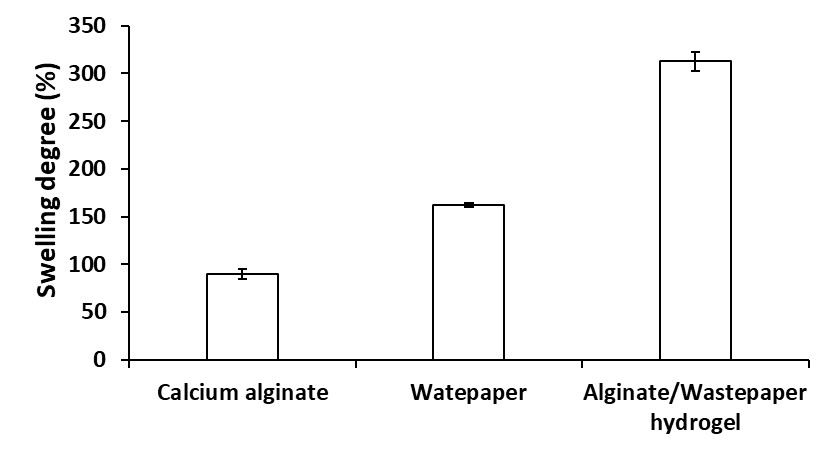


**Fig. S1:** Swelling degree of the developed alginate/wastepaper hydrogel in relation to calcium alginate and wastepaper alone. Experiment was conducted at 25 ℃ in distilled water for 60 min. Values are means ± standard deviation.

**
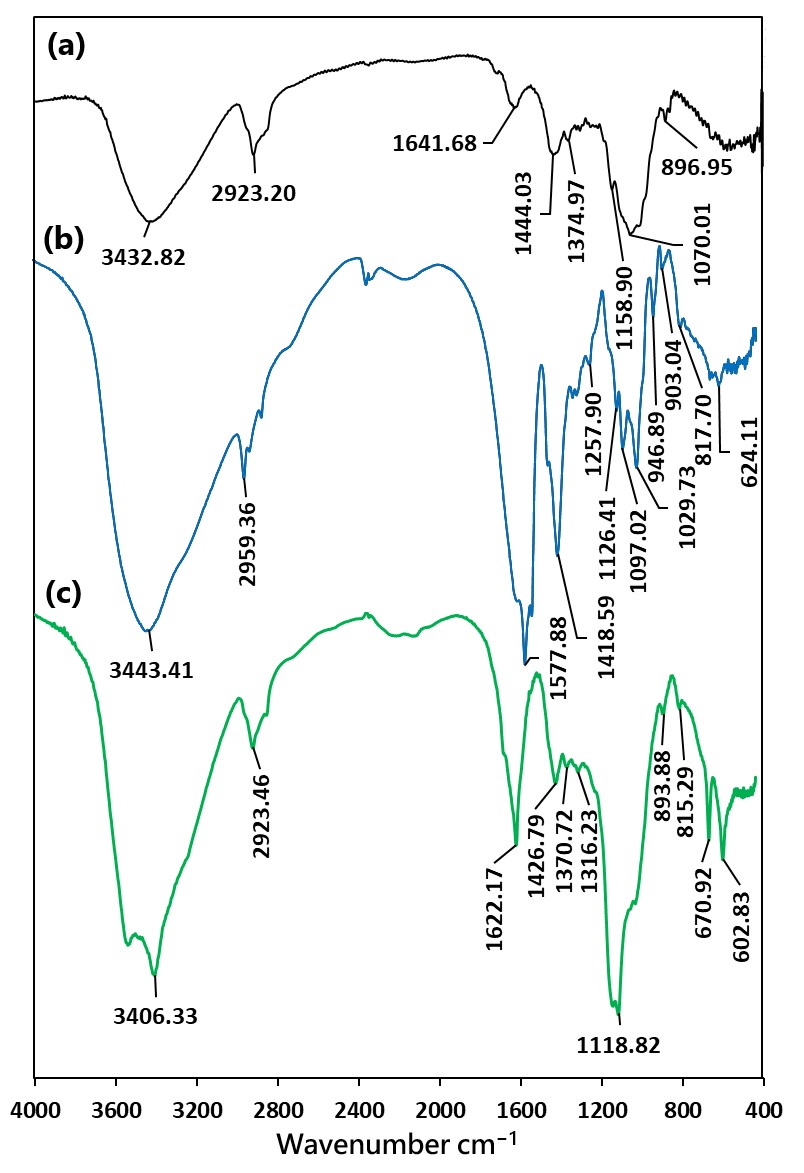
Fig. S2:** FT-IR spectra of (a) wastepaper, (b) alginate from *S. latifolium* biomass, and (C) alginate-wastepaper hydrogel.

**Table S1:**

Kinetic modelling of water absorption by the alginate-wastepaper hydrogel based on Pseudo 1^st^ order (PFO) and pseudo 2^nd^ order (PSO) and thermodynamics’ parameters.

| **Temp.**  **(ºC)** | **PFO** | | |  | **PSO** | | |  | **Thermodynamics** | | | |
| --- | --- | --- | --- | --- | --- | --- | --- | --- | --- | --- | --- | --- |
|  | **k_1_** | **R^2^** | **%ARE** |  | **k_2_** | **R^2^** | **%ARE** |  | **ΔGº** | **ΔHº** | **ΔSº** | **R^2^** |
| **25** | 0.063 | 0.939 | 20.46 |  | 0.022 | 0.939 | 20.65 |  | −1.27 | −37.66 | 131.42 | 0.938 |
| **35** | 0.132 | 0.978 | 12.91 |  | 0.027 | 0.939 | 17.13 |  | −3.28 |  |  |  |
| **45** | 0.224 | 0.862 | 12.27 |  | 0.059 | 0.828 | 14.35 |  | −4.61 |  |  |  |

k_1_: 1^st^ order rate constant (min^−1^), k_2_: 2^nd^ order rate constant (g g^−1^min^−1^), R^2^: coefficient of determination, %ARE: average relative error, ΔGº: Gipp’s free energy (kJ mol^−1^), ΔHº: enthalpy (kJ mol^−1^), ΔSº: entropy (J mol^−1^K^−1^).
